# Embodiment and enculturation: the future of architectural design

**DOI:** 10.3389/fpsyg.2015.01398

**Published:** 2015-09-16

**Authors:** Harry F. Mallgrave

**Affiliations:** College of Architecture, Illinois Institute of TechnologyChicago, IL, USA

**Keywords:** embodiment, enculturation, evolution, architecture, social cognition

## Abstract

A half-century ago the Dutch architect Aldo van Eyck encouraged designers to think about “space and time” not as abstractions in themselves but rather as cultural events better approached through the medium of “place and occasion.” Van Eyck made this point on the basis of his own travels and through his extensive readings in cultural anthropology, and his prescience is only now acquiring the credibility that it deserves through the work of a multitude of interdisciplinary researchers. Phenomenologists argue that we are embodied organisms-acting-within-environments, and these inhabiting abodes are constructed of both material and cultural dimensions. We are thus preeminently social in our range of self-consciousness, and intensely ceremonial in every facet of our being. Evolutionary psychologists and anthropologists are currently locating the origin and development of our most basic social behaviors far in our pre-human past; neuroscientists are today modeling our social circuits in the deepest reaches of our brains. Architecture would gain much from an updated cultural theory grounded in these new models of human existence.

In two semesters of the 1979 and 1980 academic years I had the good fortune to work as a teaching assistant to the Dutch architect Aldo van Eyck, a visiting professor at the University of Pennsylvania. I assisted him in the design studio and also accompanied him on a number of his outside talks. He was at that time a harsh critic of the historicist and formalist tendencies of the incipient postmodern movement, which he countered with a strong dose of anthropological fervor—encouraging architects not to think of design in such abstract terms as space and time, but rather in more social or humanistic terms as “place” and “occasion” (Van Eyck, 1976). His position, which he reiterated throughout the 1970s, was how deeply rooted in our humanity is our need for social and communal aesthetic expression and how an understanding of human culture should inform our design imaginations.

Van Eyck's advice, however, was spoken into the winds of a gathering post-structural storm, in which a widespread “incredulity toward metanarratives,” cultural or otherwise, was sweeping away the humanist footings of the past. By the end of the 1980s architectural theory was wavering between the decentered abstractions of deconstruction on the one hand and the new formalism that software-based technologies on the other. Now precluded from making any cultural statements, many designers surrendered their sketch pencils to the parametric logic of the machine. In a flurry of polemical writings a new age was proclaimed, and in the two decades since the fall of this postmodern semiotic, architectural theory has still not recovered its footing. In fact in many areas of practice today one can make the case that theory has ceased to exist altogether. Let me begin then by posing a very basic question: Can architectural theory ever again reconcile itself with the idea of cultural theory?

## Cultural theory yesterday and today

Over the last half-century cultural theory has in itself also undergone a radical transformation. The “social facts” of Durkheim, or the efforts of Weber to align ethical viewpoints with economic systems (strategies that remained cogent well into the 1960s), have today lost their viability. The thicker cultural webs of symbolic anthropology—Geertz's definition of culture as “extragenetic, outside-the-skin control mechanisms”—have too lost much of their fascination (Geertz, 1973). The underlying premise to all such systems was that while humans may have been born into the world as biological organisms, their (blank slate) characters and behaviors were largely shaped by cultural forces.

Such views are rarely proffered today, and the most important reason for their decline has been the remarkable advances within the biological sciences over the last few decades. Yesterday's debates over “nature vs. nurture” have given way to coevolutionary and enactive models in which genes and culture can no longer be considered in isolation. The sequencing of DNA alone allows us to read our evolutionary history in a way that no speculative or archaeological approach in the past could ever have imagined. One realm of biological theory known as niche construction postulates that just as we alter our physical and cultural environments, so do these changed environments alter the genetic structures and behavioral patterns of who we are. Architects, the designers of our built environments, should be taking notes.

As the formerly discrete social sciences were themselves becoming enhanced through interdisciplinary research, progress was being made along a number of related fronts. Only a few decades ago it was the consensus of many paleoanthropologists that *Homo sapiens* underwent a major cognitive breakthrough around 50,000 years ago, resulting in such things as cave paintings, complex language, and other symbolic forms of cultural transmission. Today we take a much longer view of our ancestry and with good reason. For if we broaden our lineage out to several million years and study the patterns of human behavior, we gain a quite different perspective of who we are today.

Those footprints of “Lucy” (*Australopithecus afarensis*) recorded in the volcanic ash of Tanzania 3.6 million years ago already depict a semi-erect primate who had moved away from the tropical forests of our great-ape cousins and began to forage in the savannahs of East Africa. With the appearance of *Homo erectus* around two million years ago, we have a fully bipedal species with a physical stature and body proportions similar to our own. He eventually learned to construct his housing and organize his village environment (Mania and Mania, 2005). Moreover, his social behavior was strikingly different from earlier species. Not only did he have a substantially larger brain than pre-*Homo* species but he hunted in larger groups over much greater distances of time and space, which demanded communication and coordination skills much beyond those of apes. In making the transition to the use of fire and cooked foods, he may well have practiced imitation, laughter, and other aspects of what Merlin Donald has called mimetic culture (Donald, 2001).

If we turn to *Homo heidelbergensis*, who emerged in Africa sometime after 800,000 years ago, we find a species with behaviors similar to our own. Again their group activities and social communities expanded in size and range, while anatomical changes in the vocal cord and ear canals announce the rudiments of speech. With the widespread use of communal hearths around 500,000 years ago, it is likely that we have the cultural appearance of music, song, and dance—domains formerly considered to be the exclusive purview of humans. Both the Neanderthals and modern humans likely descended from the *Heidelbergs*, and the paltry 200,000-year evolutionary timeline of *Homo sapiens* now seems little more than icing on an evolutionary cake that had been baking for several million years.

## Social cognition

One of the problems with archaeological models of a quarter of a century ago was that the principal measuring rod for human cognitive development was the so-called “tool kit,” the technology of hand axes and points. And when one considered the paltry development of the Acheulean tool kit of *Homo erectus* (employed over the first 800,000 years of his evolution), one might conclude that there was little cognitive development over this timeframe. Such a deduction would have been tenable but for one nagging fact: the cranial capacity of *Homo erectus* was nearly double the size of his pre-*Homo* predecessors. What evolutionary factors could possibly explain why an organ (the brain), which consumes 20% of the body's metabolic output, would enlarge itself so disproportionally and at such a high nutritional cost to the rest of the body? Conventional evolutionary theory offered no satisfactory explanation.

Only toward the end of the last century did a series of hypotheses begin to be put forth. In studying the cognition of great apes alongside the social development of human children, the evolutionary anthropologist Michael Tomasello pointed out that humans are unique to other primates in one important regard, which is the extent of our social cognition. Early in our evolutionary history, he argues, we initiated the process of cumulative cultural evolution or the ability to take creative inventions and pass them down to succeeding generations. The reason that we were able to create this cultural “ratchet effect” was because we developed one social skill that great apes did not, which was the ability to see other members of our species as intentional beings with mental lives similar to our own (Tomasello, 1999, p. 5). This unique skill began to be cultivated early in our evolution, around the start of the *Homo* genus, but it greatly accelerated with the *Heidelbergs*, who likely practice such social skills as pantomime, simple representation, self-monitoring, inference, and a willingness to seek conformity with others in social groups (Tomasello, 2014, p. 48–66). In other words, just as we change the aspects of our cultural context, so does our ever-changing cultures alter our cognitive structures.

Also in the 1990s the evolutionary psychologist Robin Dunbar put forth his “social brain hypothesis,” which proposed that the cultural complexity of our ever expanding social networks (our families, friends, enemies, clans, and larger social alliances) necessitated the expansion of our cognitive powers in order to cope with this new social reality (Dunbar, 1998). Whereas, the tenet of a correlation between big brains and the size of social communities is generally accepted, there remains much discussion about the cause—that is, of whether meta-representational models such as mind-reading (theory of mind) are needed to explain social cognition or whether cognitive complexity might be more parsimoniously explained through embodied and distributed processes of cognition built into our perceptual activity (Barret et al., 2007).

Such challenges have been assisted by two other developments of the 1990s: a more embodied understanding of emotion and the discovery of mirror mechanisms. In the first regard there was there were the pioneering efforts of Jaak Panksepp and Antonio Damasio, both of whom viewed emotion less as a psychological state of mind, the antithesis of logical reasoning, and more as reason's very biological foundation (Damasio, 1994). Emotions are “affect” or electrical/chemical programs that shape or shortcut the way in which we perceive the world, basically as pleasurable or non-pleasurable events (Panksepp, 1998). In its simplest definition, emotion is the pre-reflective response of an organism to a stimulus, and translated into architectural terms it can be described as the pre-reflective response of the human organism to the built environment. In this way architectural design, similar to human cognition in general, is fundamentally emotional in its enactive properties.

The discovery of mirror mechanisms in the early 1990s has similarly provided new insights into how we perceptually engage with the world. They were first discovered in macaque monkeys in a lab at the University of Parma and within a few years humans were also shown to possess such systems, although in a more complex way (Rizzolatti and Sinigaglia, 2008). Mirror mechanisms are sensorimotor circuits that fire not just when we perform an action, but when we see or hear someone else performing an action, such as playing the piano or lifting a tea cup. Effectively, parts of our sensorimotor circuits respond as if we were performing the action, excluding those motor circuits by which we would actually perform the action. The process has been called “embodied simulation” and it is the reason why we enjoy watching an athlete or a ballet dancer (Gallese, 2005; Gallese and Gattara, 2015).

Around the turn of this century the issue arose as to whether mirror mechanisms might also explain how or why we are so facile at reading or sharing the emotions of others—that is, how in seeing a friend we know immediately her state of mind, as if we share an empathic accord with her. We now know that through neurological mechanisms we internalize and simulate the emotions of others, although the extent to which mirror mechanisms are learned is still being debated today (see, for instance, Catmur et al., 2007). Human empathy, it seems, possesses deep evolutionary, biochemical, and neurological underpinnings, activating the cortical and limbic areas, brainstem, autonomic nervous, and endocrine systems. These mirror circuits at the same time underscore just how basic empathy or sociality is to our human natures. We do not become social simply through cultural training; we are born social in the non-nativist sense that we come into the world with the neural capacity to construct affordances through our social engagement with the world. What has also emerged from this research is a very tidy explanation of how we have distinguished ourselves from our primate ancestors. We took the mirror and other neural mechanisms already present in our primate lineage and—over the course of a few million years—bridged the cognitive, sensorimotor, and somato-visceral dimensions of our evolution.

## A phenomenological model for architectural research

As the many implications of all of these events began to become known around the turn of the present century, it is quite understandable how and why philosophy itself underwent a critical upheaval during the same period. We are, as the phenomenologist Edmund Husserl noted many years ago, “animate organisms” sensorialy and emotionally attuned to our surroundings, yet the predominant interest of philosophy throughout the twentieth century was its almost exclusive focus on the rational aspects of our being. Cognition, within philosophical literature in fact, has often been reduced to this single capacity: the exercise of the Cartesian *cogito*. Theories of embodiment first appeared as a way to correct this dualistic bias, but only now are we realizing the full extent to which the body actually shapes our thinking. We are also beginning to see, as the philosopher Evan Thompson has recently noted, that the nervous system, body, physical, and cultural environments are dynamically integrated with each other on multiple levels, and how, as a consequence, the developmental processes of human life reconstruct themselves anew in each generation in response to ever-changing genetic, cellular, social, and cultural factors (Thompson, 2007). The brain, the body, and the environment are in effect codetermining of each other and therefore coevolving.

In a paper of 1998, “Radical embodiment: neural dynamics and consciousness,” Thompson and Francisco Varela provided a formula for considering these issues by noting that there are three realms or dimensions of embodiment in higher primates:

1. cycles of organismic regulation of the entire body;
2. cycles of sensorimotor coupling between organism and environment;
3. cycles of intersubjective interaction, involving the recognition of the intentional meaning of actions and linguistic communication (in humans).

                       (Thompson and Varela, 2001)

Whereas such a model was proposed for philosophical analysis, it is a model that can be readily transposed into areas of research relating to the practice of architecture:

1. Human biology and the built environment (homeostatic adjustments to the built environment);
2. Perceptual and aesthetic experience of the built environment (the dynamics of sensorimotor coupling);
3. Sociocultural nature of the built environment (intersubjective interactions).

## Human biology and the built environment

The form and muscular-skeletal characteristics of our bodies, our nervous, and perceptual systems, and the many facets of our affective and cognitive networks—all have evolved over the last 2.4 million years (limiting ourselves to the species *Homo*) largely within natural terrains. This realm will consider the functioning of the human organism within the built environment, our altered terrains and present-day niches. Central to this description are basic descriptions of the human nervous system, the characteristics of its growth and development, the perceptual systems by which we orient and navigate ourselves with respect to our surroundings, and the impact of factors such as sensory enrichment and deprivation on our health and happiness.

## Perceptual and aesthetic experience of the built environment

With this dimension of embodiment we can take some new and bold initiatives, because neuroimaging technologies now allow us to explore with considerable precision the human experience of the built environment. For instance, we can now begin to study human responses to various materials (steel, glass, concrete, wood), the dynamics of personal and peripersonal space, our biological responses to certain spatial settings, human responses to particular forms, colors, proportions, textures, light, and vegetation—in short the many enactive variables that compose the built environment. The goal here, it must be emphasized, is not to prescribe guidelines or presume some kind of one-size-fits-all design, but to underscore the intensity and wealth of experiences that architecture can provide and present this information as a challenge to the designer. When this research is directed at larger urban scales, it will allow us to discuss in an informed way how our cities might be conceived in a more humane way.

## Sociocultural nature of the built environment

It is at this level that we can introduce and expand upon current research in social empathy and social neuroscience, and align architectural theory with the premises of contemporary gene-culture theory. How we do so should be debated, but again we should stress that we are biological organisms with certain basic needs. Architecture does not exist to coax human behavior (our cities are filled with failed social experiments or “solutions” of the past) but to let it unfold in a natural and dignified way, attuned to our sociality. Architects have often invoked such terms as “atmosphere” in relation to design—that is, how a fireplace within a room, or a pleasant view into a plaza or garden, informs the mood of those experiencing it. From a sociocultural perspective, one might define architecture as the psychological setting for a mood, or, in the words of van Eyck, as the making of a “place” and “occasion” for social ceremonies and aesthetic rituals. Many years ago Gottfried Semper referred to the hearth as the “moral element” of architecture, and in his cultural exposition of this theme he described the “sacred flame,” later socially housed within the festival apparatus, as “the *motive* for the *permanent monument*, which is intended to proclaim to future generations the solemn act or event celebrated.” At this point he inserted his well-known footnote on the “dressing and the mask” and cautioned the architect not to “blurt out” the theme of the artistic creation, but to mask it in an aesthetic or playful way (Semper, 2005, p. 438–439). Similarly—if indeed our human ancestors engaged in laughing, singing, and dancing around a fire as early as one million years ago, we should now recognize and respect ourselves for the singers, dancers, and masked personas we really are. Only in this way will our new knowledge of ourselves, far from being reductive, allow us to explore and reclaim the multiple dimensions of our distant humanity.

## Conflict of interest statement

The author declares that the research was conducted in the absence of any commercial or financial relationships that could be construed as a potential conflict of interest.
